# Lost diagnoses? A multi-year trajectory of patients with childhood ADHD in the criminal justice system in Switzerland

**DOI:** 10.3389/fpsyt.2024.1403618

**Published:** 2024-06-06

**Authors:** Helen Wyler, Moritz van Wijnkoop, Alexander Smith, Wolfgang Retz, Michael Liebrenz, Ana Buadze

**Affiliations:** ^1^ Department of Forensic Psychiatry, University of Bern, Bern, Switzerland; ^2^ Faculty of Behavioural Sciences and Psychology, University of Lucerne, Lucerne, Switzerland; ^3^ Institute for Forensic Psychology and Psychiatry, Saarland University Medical Center, Saarland University, Homburg, Germany; ^4^ University Medical Centre, Johannes Gutenberg University Mainz, Mainz, Germany; ^5^ ADHD Specialty Clinic, Department of Psychiatry, Psychotherapy and Psychosomatics, Psychiatric Hospital, University of Zurich, Zurich, Switzerland

**Keywords:** ADHD, forensic psychiatry, offending, comorbidity, expert witness assessment, treatment, recidivism, criminal justice system

## Abstract

**Background:**

Attention-deficit/hyperactivity disorder (ADHD) is prevalent amongst offenders, increasing risks for aggressive and delinquent behaviors. Since ADHD and its symptoms can persist into adulthood, accurately diagnosing and maintaining diagnoses in offenders is crucial to ensure appropriate treatment and reduce recidivism.

**Methods:**

This study employed a retrospective longitudinal design to investigate ADHD amongst adult offenders with a confirmed diagnosis of ADHD during childhood or adolescence at a Swiss forensic outpatient clinic between 2008 and 2021. N = 181 patient files were reviewed, including forensic expert witness assessments and treatment reports. We charted the adulthood trajectory of patients with a confirmed childhood/adolescence ADHD diagnosis, examining the course of their diagnoses.

**Results:**

Of 181 patients, evidence indicated that 12 (7%) had an ADHD diagnosis in childhood/adolescence. In 1 (8%) of these 12 cases, the diagnosis was maintained throughout the observation period. For 4 patients (33%), a diagnosis was given in the first forensic psychiatric expert witness assessment in adulthood but subsequently dropped. In another 4 cases (33%), the diagnosis was dropped in adulthood but later re-assigned, whereas in 3 cases (25%), the diagnosis was discontinued throughout the observation period. In 50% of cases with a diagnostic change, the discontinuation of an adult ADHD diagnosis coincided with a newly diagnosed personality disorder (or vice versa).

**Conclusions:**

Our findings highlighted considerable inconsistencies in the assignment of adult ADHD diagnoses amongst offenders. Whilst ADHD remission in adulthood occurs, the diagnostic variability in our results warrants detailed scrutiny. One possibility is that ADHD has similar fluctuations to conditions like depression, as argued elsewhere. Equally, diagnoses may become “lost”, meaning they are not given even when applicable and replaced by other diagnoses. Additionally, residual symptoms may remain but beyond the diagnostic threshold. This is significant because untreated ADHD can increase re-offending risks and adverse health outcomes.

## 1 Introduction

As a neurodevelopmental disorder associated with inattention and/or hyperactivity/impulsivity, attention-deficit/hyperactivity disorder (ADHD) frequently leads to functional impairments in affected individuals (e.g (1, 2).,). ADHD often develops due to a combination of genetic and environmental determinants, with varying symptoms that generally decrease with age (3, 4). However, some patients experience severe symptoms continuously and diagnoses may persist throughout adolescence or adulthood (4). According to research, the proportion of individuals with an ADHD diagnosis as children who meet full diagnostic criteria into early adulthood varies from 17% to 50%; with 50% to 90%, the proportion of individuals who exhibit at least residual symptoms in early adulthood is estimated to be even higher (5–7).

Whilst ADHD prevalence rates for the general adult population typically range between 2% and 3% (8, 9), considerably higher figures have been observed in specific subpopulations, particularly in forensic settings. For incarcerated adults, meta-analytic findings indicate an average prevalence rate of 26% (10, 11), which is a nearly ten-fold increase compared to the general adult population. A nationwide study in Denmark reported that the risk for convictions associated with ADHD increased by 60%, even after controlling for other known criminogenic risk factors (12). This suggests that ADHD may be a risk factor in and of itself rather than just co-occurring with other risk factors. This notion is supported by other research, although the influence of comorbid mental disorders, such as anti-social personality disorder (ASPD), is not yet fully understood (13, 14). For individuals with ADHD with a previous conviction, an elevated risk of recidivism has been observed (13, 15, 16). Equally, ADHD not only increases the probability of criminal behavior and recidivism, but also the possibility of intramural offences. For instance, intramural offenders with ADHD were found to be eight times more likely to exhibit aggressive behavior than inmates without ADHD (17).

Notably, research suggests that it may be possible to mitigate these increased (re)conviction risks through psychopharmacological treatment for underlying ADHD. An investigation analyzing Swedish national register data on 25,656 ADHD patients showed that medication led to a 32% reduction in criminality for men and 41% for women, compared to non-medication periods (18). Analogous results were apparent in a more recent study by Mohr-Jensen et al., where criminal outcomes were significantly higher but reduced during periods of taking ADHD medication in a sample consisting of 4,231 individuals with ADHD compared with a control group from the general population (12). Furthermore, psychostimulants have been reported to be associated with a substantial risk reduction for violent reoffences, as noted in a cohort study that focused on 22,275 people released from prison in Sweden (19). Moreover, Dalsgaard et al. documented the potential protective benefits of psychopharmacological treatment against criminal behaviors in an investigation into the causal effects of this treatment amongst children with ADHD in Denmark (20). Although pharmacoepidemiologic studies are open to confounding factors, the effects persisted even after controlling for these confounders. For instance, the discontinuation of other medication instead of ADHD medication did not yield the same effect. This reduces the probability that supportive environments, personal motivations, and other variables related to the use of medication were relevant factors in the association between ADHD medication and crime rates (18, 19). Additionally, the fact that violent reoffending, rather than other forms of reoffending, were linked to ADHD medication supports a direct effect (19). By contrast, the extent to which psychopharmacological treatment in correctional facilities reduces ADHD symptoms and results in other positive (non-violence or crime-related) outcomes is an ongoing question; the few primary studies that exist to date yield an inconsistent picture (21).

While the evidence discussed underlines the importance of correctly identifying and treating ADHD in adult offenders, ADHD is known to be notoriously under- and misdiagnosed in offender populations (22–24). In an investigation in a Swiss prison, nearly 13% of 158 participants met the cut-off criteria in a self-reported ADHD screener, whilst only 2% had a clinical diagnosis of ADHD in their medical files (24). Significantly, no participants in this sample were receiving treatment for ADHD (24). Elsewhere, a study on a Dutch forensic outpatient sample noted that an ADHD diagnosis had previously been missed in 56% of cases where forensic patients received a diagnosis through referral (25). Similarly, two studies involving individuals in prison in Australia and the United Kingdom respectively showed that of those who met the criteria for an ADHD diagnosis, only between 6.7% of 30 individuals (Australia) and 18.8% of 390 individuals (United Kingdom) had been diagnosed prior to the study assessment (26, 27).

In sum, being correctly diagnosed with ADHD is pivotal for affected individuals, especially offender populations. A correct diagnosis of ADHD, which is maintained during adulthood (where appropriate), thus benefits both the individual and wider society, informing appropriate therapeutic approaches (23). Within this context, the present study aims to add to the wider understanding of the trajectories of childhood ADHD diagnoses in criminal offenders who received court-mandated treatment in a forensic outpatient clinic. Particularly, we were interested in (1) what portion of a forensic outpatient sample had received a diagnosis of ADHD during childhood, and (2) how the diagnosis evolved during a multi-year interagency treatment process (i.e. at what point(s) the diagnosis was maintained or discontinued).

To that end, we analyzed whether an ADHD diagnosis was present in expert witness assessments, specific ADHD assessments, and psychotherapeutic treatment reports during the period of observation. We were also interested in comorbid diagnoses and whether the on- or offset of an ADHD diagnosis was accompanied by changes in other diagnoses. The results are expected to yield broader insights into whether ADHD diagnoses persist into adulthood in offenders and, if not, reveal potential reasons why diagnoses may be discontinued.

## 2 Methods

### 2.1 Study design

We employed a retrospective longitudinal cohort study design to chart the trajectories of individuals who had been receiving psychotherapeutic treatment at the Bern Forensic Outpatient Clinic (BFOC) at the University of Bern between 2008 and 2021. The BFOC provides treatment for offenders and is unique in German-speaking Switzerland for its permanent mandate from the Canton of Bern (28). The patient files included information from their first expert witness assessment as an adult to the last relevant document available (i.e., expert witness assessment, ADHD assessment, forensic-psychiatric/psychological treatment report), before discharge from the BFOC.

### 2.2 Sample

The full sample consisted of the data of *N* = 181 individuals. These patients were ordered to partake in a therapeutic measure as part of their sanction or by the correctional authorities. In the Swiss forensic system, offenders can be treated under a therapeutic measure per Article 59 (inpatient treatment), Article 63 (outpatient treatment), or on a voluntary basis [for more details see (29)]. At the BFOC, treatment sessions are conducted by forensic psychiatrists and psychologists; social workers may provide additional support, but they do not engage in psychotherapeutic treatment. Visits to the clinic for offenders are mandatory as part of the therapeutic protocol, especially given the correctional environment. The frequency of these visits is dependent on the specific needs of the patients, ranging from weekly appointments during initial treatment or acute phases to bi-weekly or monthly visits as the therapeutic measure nears completion. The diagnoses are monitored and discussed by a team of psychologists and psychiatrists. Changes are decided upon in a consensual manner, with the forensic psychiatrist having the final decision and being responsible for reporting back to the Office for the Execution of Penal Sentences and Justice (*Amt für Justizvollzug*). If the therapeutic team suspects a change may be needed to the existing diagnoses, it can also refer the patient for assessments, such as an ADHD assessment.

To be included in the main analyses, the following inclusion criteria had to be met: (1) sufficient materials available (including at least one forensic expert witness assessment, as these usually contain information on the case history that allows establishing the presence of an ADHD diagnosis during childhood or adolescence), (2) a clear indication of an ADHD diagnosis during childhood or adolescence, and (3) treatment at BFOC concluded by the end of 2021. We excluded those cases where treatment at the BFOC remained ongoing since it would not have been possible to trace their full trajectories; this could have led to the omission of adult ADHD diagnoses that were being discontinued or newly made.

The same process of identifying cases for the main analysis was applied to all cases of patients registered at the BFOC between 2008 and 2021 who had concluded their treatment by the end of 2021 (and whose files were available). Initially, all available documents were incorporated in an automated search for keywords related to ADHD using the analytical software, NVivo, to identify the cases where ADHD may have been relevant. The keyword list encompassed a total of 36 words related to the disorder name (e.g., ADHD), symptoms (e.g., inattentive), diagnosis (e.g., F90.0), and medication (e.g., methylphenidate). The documents included court files, documents from the authorities, forensic expert witness assessments, medical reports, psychotherapeutic treatment reports, enforcement reports, internal case documentation and case overviews, medication sheets, ADHD assessments, reports from residential escorts, and other documents (e.g., emails, disability insurance documents).

In the second phase, cases that were identified as potentially relevant were reviewed individually by two members of the research group. Cases that did not contain sufficient evidence for the presence of childhood or adolescent ADHD were excluded. If, for instance, a patient reported to the expert witness that they were informed at school that they had ADHD but that they did not receive a classified diagnosis, the case was not included. Equally, if available information was contradictory, such as several experts with strongly divergent opinions about a possible ADHD diagnosis when the patient was a child or adolescent, the case was not incorporated in the main analyses. In addition to those cases with a verified diagnosis of ADHD in childhood or adolescence, we also included cases with the diagnosis of a so-called psycho-organic syndrome (*Psychoorganisches Syndrom* – POS). Although ADHD was separated from the clinical concepts of POS or minimal cerebral dysfunction in the 1980s (30), the term POS has persisted. In Switzerland, for instance, this classification was and still is relevant in the context of Swiss disability insurance. According to Berger et al., the POS diagnosis differentiates simple ADHD presentations from ADHD with persisting cognitive deficits for Swiss health insurance purposes (31).

**Figure 1:**
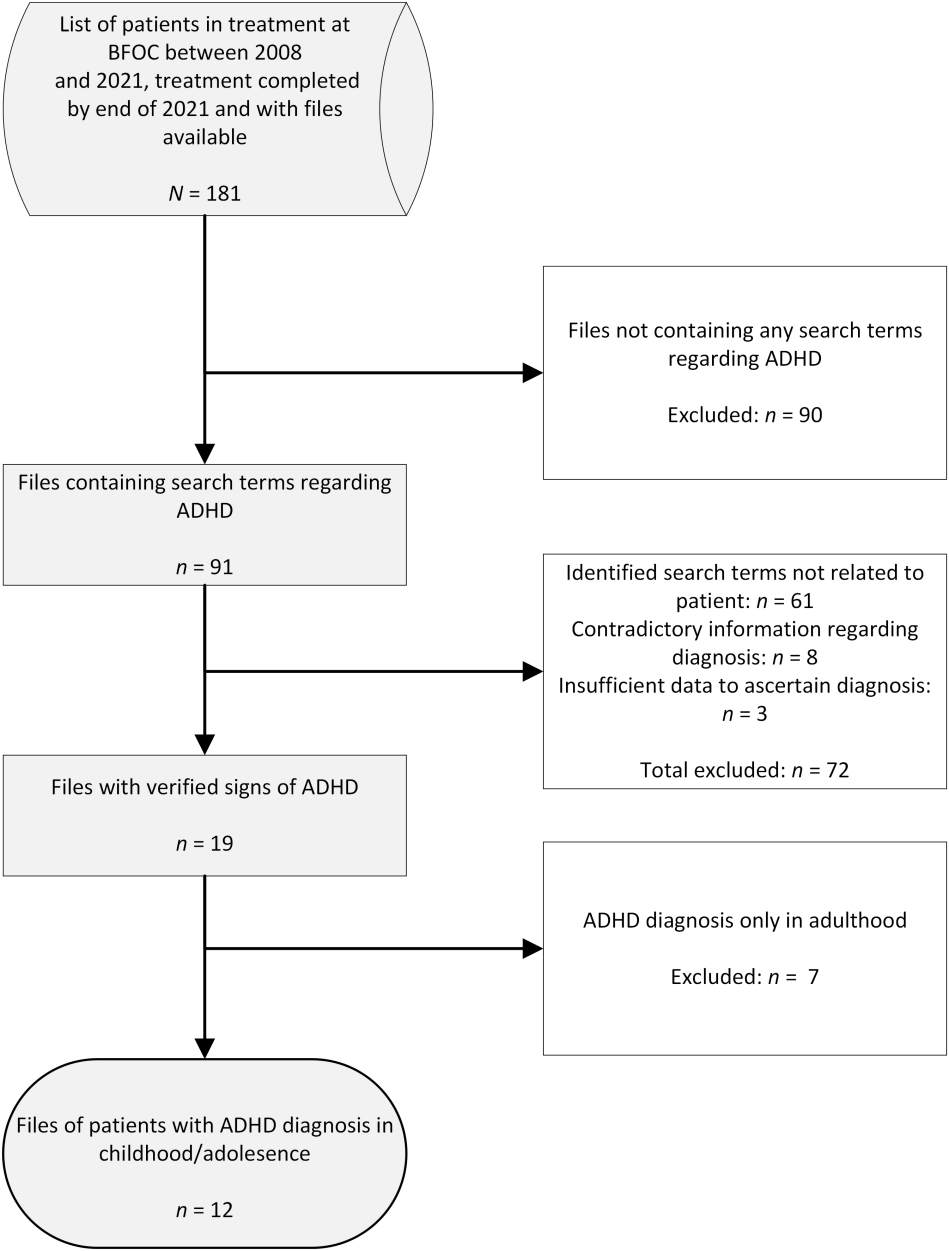
Flow chart of case selection.

Following this methodology, the resulting dataset contained N = 12 patients where clear indications were found for the presence of an ADHD diagnosis in childhood or adolescence. An overview of the case selection is shown in **Figure 1**. The study was approved by the Ethics Commission of the Canton of Bern (ref.-no. 2022-02113).

### 2.3 Included documents

We considered three types of files from which diagnostic information was extracted. First, we included forensic expert witness assessments, which provide diagnoses according to one of the recognized classification manuals (typically ICD). In some cases, these assessments also provided details on previous expert witness reports, which included contemporaneous diagnoses. To ensure a comprehensive overview, we reviewed any forensic-psychiatric expert witness assessments made in the context of a criminal trial as a separate data point in the timeline for each case, even if our files did not include the original report. If at the time of the report an individual was below the age of 18, the report was not included. In Switzerland, initial expert witness assessments (*Erstgutachten*) are usually concerned with assessing legal culpability, whether a psychiatric diagnosis existed that was relevant in the context of the crime, legal prognosis, and the question of whether court-mandated therapeutic treatment would contribute to reduce the risk of re-offending. Equally, in Switzerland, expert witness statements on progression (*Verlaufsgutachten*) may be requested to provide information on the effectiveness of a therapeutic measure, legal prognosis, and/or the expert’s opinion on individual psychiatric diagnoses.

Secondly, we coded the outcome of ADHD assessments conducted by an expert witness psychologist using psychometric tests, such as the Homburg ADHD scales for adults (HASE), which is a standardized German scale (32, 33). Assessments that only reported measures of selective and sustained attention [e.g. using the d2 Test of Attention (34)] were excluded, as this is not sufficient to assess ADHD. In Switzerland, psychiatrists typically make diagnoses in the context of expert witness statements for criminal law rather than psychologists. Thus, the ADHD assessments only comment on the likelihood of ADHD having persisted into adulthood. For the avoidance of doubt, whenever the disorder persisted with at least a high probability, the diagnosis of ADHD was coded as present.

Thirdly, we analyzed all forensic-psychiatric/psychological treatment reports (henceforth referred to as treatment reports) from the BFOC and other institutions available in the files in which diagnoses were discussed. In many cases, the report repeated the diagnoses from the expert witness report. We considered these instances as the therapist accepting the diagnoses from the report at that time as they did not actively disagree. In other cases, the therapist disagreed with previous diagnoses or added new classifications and this outcome was coded accordingly. Certain treatment reports were excluded either because diagnoses were not discussed, because the diagnoses could not be coded using internationally accepted classification manuals, or because the therapist listed all the diagnoses made in previous assessments or reports without indicating what report or diagnoses, they would work from.

### 2.4 Coding

For the remaining 12 cases, members of the research team extracted relevant information from the files. This included demographic data (i.e., sex, age, nationality), the nature of the index offence, and detailed information on the diagnoses made in the three types of documents outlined above. We did not find a section where the diagnoses were clearly outlined using an internationally accepted coding system in all instances. Accordingly, these cases were verified with a senior member of the research team (who is a forensic psychiatrist with extensive expertise in the areas of forensic expert witness assessments, ADHD, and forensic-psychiatric treatment). Suspected diagnoses were not coded.

### 2.5 Analyses

Owing to the limited number of cases that remained for the in-depth analyses and the considerable differences between individual cases, no statistical analyses were conducted. Descriptive statistics were used and a visualization of individual trajectories was produced to present the findings.

## 3 Results

### 3.1 Sample description

Of the total of 181 patients, for 12 (6.6%) there was sufficient evidence to confirm the presence of an ADHD diagnosis during childhood or adolescence. All 12 patients were male Swiss nationals and 11 had been sentenced to a therapeutic measure (*therapeutische Massnahme*) by a Swiss court. For one patient, the court strongly recommended that treatment should be given based on the recommendation of the forensic-psychiatric expert witness. However, as the individual did not meet the threshold for a diagnosis of a psychiatric disorder based on ICD-10 classifications, no therapeutic measure was ordered. In descending order of frequency of crimes against the Swiss Criminal Code and Narcotics Act, the most common index offences included criminal property damage (7), theft (4), drug offences (4), (qualified) sexual assault (3), (qualified) robbery (3), grievous bodily harm (3), and actual bodily harm (3). There were also individual instances of rape, arson, kidnapping, child sex offence, and attempt at premeditated murder. See **Table 1** for further information on the sample characteristics.

**Table 1:** Case characteristics of the main sample.

	*M*	*SD*	*Range*
age at index offence	21.75	2.14	19-26
duration of the observation period	9.75	3.72	6-19
no of expert witness assessments	2.58	1.24	1-4
no of ADHD assessments	0.50	0.52	0-1
no of treatment reports	6.08	3.12	3-14

The number of expert witness assessments refers to both assessments available in full (n = 22) and former assessments that were referred to in another assessment (n = 9). The duration of the observation period refers to the number of years for which documents providing information on the diagnoses were available.

### 3.2 Trajectories

**Figure 2:**
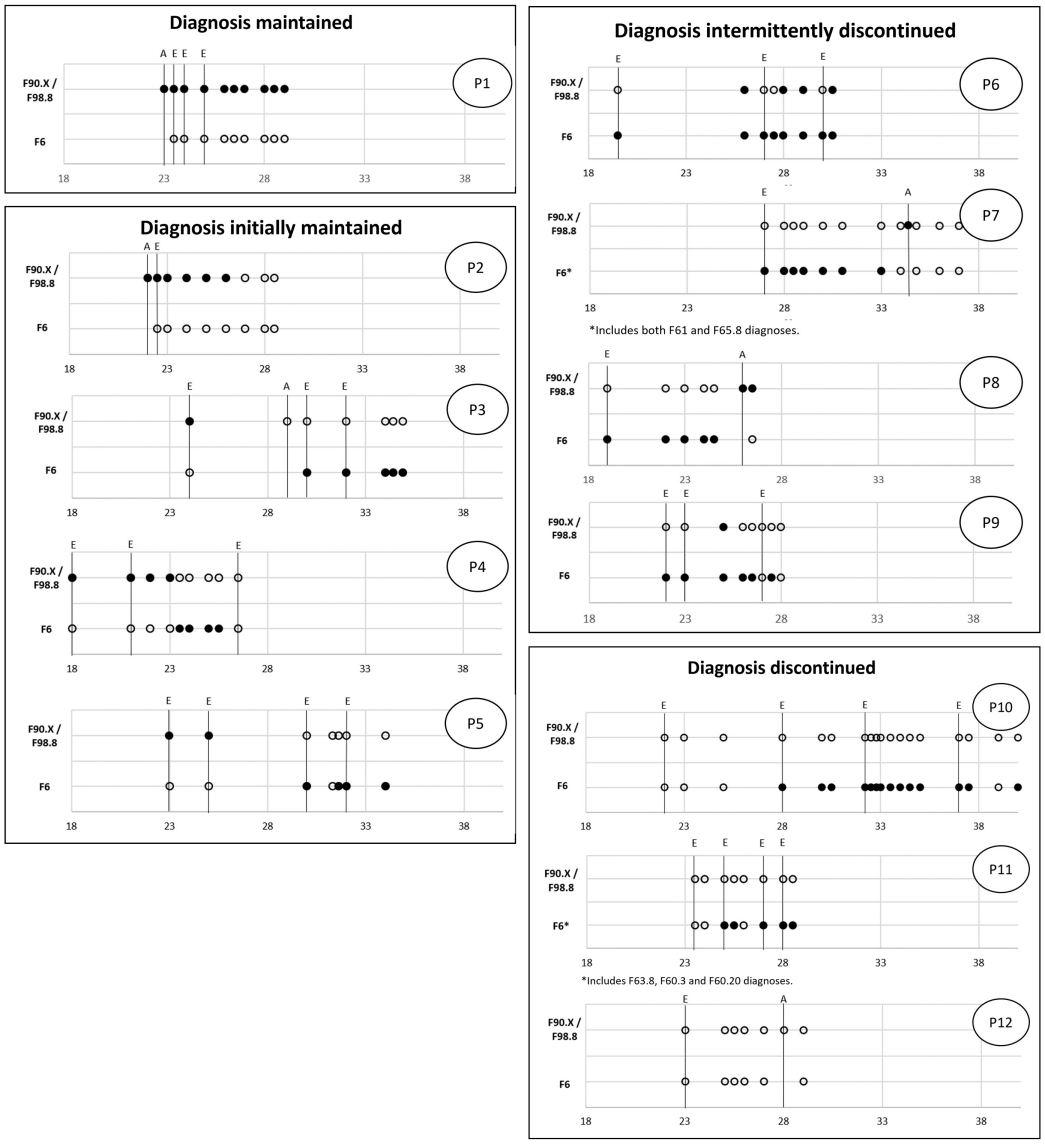
Individual courses of diagnoses in offenders who had ADHD in childhood/adolescence. A indicates an ADHD assessment, E refers to a forensic-psychiatric expert witness report, and all other assessment points are based on treatment reports. For the avoidance of doubt, all diagnostic classifications are based on ICD-10. All filled points indicate the presence of a specific diagnosis. For empty points, a specific diagnosis was not made. PX refers to the individual patient cases also referred to in the results section.

A total of 110 documents were evaluated for the diagnoses mentioned for the 12 cases included in the main analysis. These consisted of 31 forensic expert witness assessments, 6 ADHD assessments, and 73 treatment reports. The expert witness reports available in full were *M* = 60 pages long (*SD* = 35 pages), with a range of 20-179 pages. Individual trajectories were mapped for the patients who had a diagnosis of ADHD in childhood or adolescence, as displayed in **Figure 2**.

From our analysis, we observed four distinct trajectories. In one out of the 12 cases (8.3%), the diagnosis of ADHD was *maintained* throughout the full observation period (P1 in **Figure 2**). In four cases (33.3%), the diagnosis was present at the first point of observation but discontinued at a later stage (*initially maintained*; P2-P5). In another four cases (33.3%), the diagnosis was not made at the first point of observation but reappeared at a later stage either continuously or intermittently (*intermittently discontinued*; P6-P9). Finally, in three cases (25.0%), the diagnosis was *discontinued* throughout the whole of the observation period (P10-P12) (P3).

For the one case in which the ADHD diagnosis was maintained throughout, we analyzed the first expert witness report for salient details. For the cases where there was at least one change in an ADHD diagnosis during adulthood (*n* = 8), we investigated in what context the change occurred. For the three cases where no adult ADHD diagnosis was made, we examined whether the possibility of adult ADHD was discussed and assessed.

#### 3.2.1 Diagnosis maintained

The ADHD assessment using HASE concluded that the patient met the criteria on all scales (i.e., inattention, hyperactivity, impulsivity, hyperactivity/impulsivity). Whilst the expert witness argued the patient had an immature personality, because of their overall ability to function well in everyday life (e.g. maintaining reliable interpersonal relationships) a diagnosis of personality disorder was not deemed appropriate.

#### 3.2.2 Diagnosis initially maintained

Where an ADHD diagnosis was initially maintained, the diagnosis was no longer upheld in an expert witness assessment in two cases. In the first case (P3), an additional ADHD assessment using HASE did not show evidence of symptoms indicating the presence of adult ADHD fulfilling all criteria. It should be noted, however, that we did not have access to the full report for this specific assessment and thus relied on information in the expert witness assessment, for which the additional ADHD assessment was made. In the second case (P5), the expert witness assessment included limited information as to why the ADHD diagnosis was no longer applicable. No diagnostic assessments were included. In both cases, the patients were newly diagnosed with a personality disorder.

Separately, for two other patients, the diagnosis was discontinued in the context of a treatment report. In one case (P2), the report stated that the problems associated with ADHD diagnosed by the expert witness were neither observed in the patient’s apprenticeship nor at school. In the other case (P4), the expert argued that what had originally been diagnosed as F90.1 should now be diagnosed as a personality disorder, given the patient’s age and since certain behavioral patterns became persistent over time.

#### 3.2.3 Diagnosis intermittently discontinued

In all cases, an ADHD diagnosis had been given during childhood or adolescence but was not made during the first forensic expert witness assessment in adulthood. For these trajectories, the following observations were apparent.

In one case (P9), the first two expert witness assessments affirmed that ADHD had not persisted into adulthood. However, in the first available treatment report, an ADHD diagnosis was listed, and the patient received psychopharmacological treatment. Another treatment report from a different facility stated that the assumption of “persisting residual symptoms” of ADHD seemed plausible. However, the next treatment report, from still another facility, focused on a cluster B personality disorder, even though ADHD-related psychopharmacological treatment was continued.

In a second case (P6), the diagnosis re-emerged in treatment reports between expert witness assessments where residual symptoms were identified at most. The first expert witness report did not report the presence of adult ADHD, whereas an additional psychiatric expert opinion diagnosed ADHD but clearly stated that it was unrelated to the offences (and, thus, not relevant from a forensic-psychiatric perspective). The first treatment report available assumed the presence of adult ADHD and a personality disorder that evolved in the context of ADHD. ADHD-related medication was prescribed. The second expert witness report identified residual symptoms insufficient to warrant a full diagnosis. The following treatment report concurred with the presentation of residual symptoms. However, two subsequent reports, provided by a different facility, refer to the same expert witness report but (either incorrectly or deliberately in the sense of a change) list ADHD as one of the diagnoses from this report. Another expert witness assessment by the same expert witness as the second report stated there were no qualitatively or quantitatively noticeable diagnostic changes to the previous assessment, whereas the final treatment report again listed ADHD as a diagnosis. The patient received ADHD-related psychopharmacological treatment throughout.

In the final two cases, ADHD assessments suggested the likely presence of adult ADHD. In one of the cases (P8), an ADHD diagnosis followed the ADHD assessment in the subsequent treatment report. In the other case (P7), the ADHD assessment, stated “[t]hese results indicate the presence of ADHD in adulthood”. This was discussed in the next treatment report but was interpreted as indicating that the patient did not have adult ADHD fulfilling all criteria.

#### 3.2.4 Diagnosis discontinued

For the three cases where the ADHD diagnosis was discontinued completely, it is not possible to identify at what point a change occurred. Accordingly, we analyzed the first forensic psychiatric expert witness assessment as this likely affected any further diagnostic considerations.

In the first case (P10), it is argued that the patient has cognitive impairments that are related to an infantile organic brain disorder. However, no ICD-10 diagnoses were made, and the possibility of adult ADHD was not discussed.

In a second case (P11), whilst the result of the Wender-Utah Rating Scale (Ward et al., 1993) did not reach the cut-off for childhood ADHD, the question of childhood ADHD could not be settled as the observer rating provided to a parent was not returned. It remains unclear whether this was why adult ADHD was not discussed as part of the diagnostic assessment cited in the expert witness report, although it was noted that the diagnostic tests did not show any impairment of the patient’s attention or concentration performance. The expert concluded that the patient’s childhood ADHD had developed into impulsive personality traits but that, otherwise, the patient had mostly “outgrown” their ADHD.

For the last case (P12), the expert stated that some information contained in a discharge report from a pediatric psychiatric service may be indicative of ADHD (at the time). However, the expert further noted that if the diagnostic criteria for ADHD had been met at the time of the evaluation, the institution would have likely diagnosed the patient with ADHD. No further discussion on the topic was evident. A later ADHD assessment concluded that whether the concentration problems were related to ADHD was questionable, particularly because the individual indicated in their self-report that these issues did not result in personal distress.

### 3.3 Other diagnoses

Finally, we analyzed the other psychiatric diagnoses that patients received. The presence and absence of all diagnoses and comorbidities for each patient are presented in the supplemental online materials. **Table 2** presents a summary of the occurrence of other diagnoses. A diagnosis was counted if it was made at least once. For the cases with adult ADHD, we report the comorbidities, that is, diagnoses that were present at least at one assessment point at which an ADHD diagnosis was made as well.

**Table 2:** Frequency of different psychiatric diagnoses.

	% of all cases (*N = 12*)	% of cases with adult ADHD (*n = 9*)	% of cases without adult ADHD (*n = 3*)
F0: Mental disorders due to known physiological conditions	8.3% (1)	11.1% (1)	0% (0)
F1: Mental and behavioral disorders due to psychoactive substance use	66.7% (8)	66.7% (6)	33.3% (1)
F2: Schizophrenia, schizotypal, delusional, and other non-mood psychotic disorders	0% (0)	0% (0)	0% (0)
F3: Mood (affective) disorders	0% (0)	0% (0)	0% (0)
F4: Anxiety, dissociative, stress-related, somatoform and other nonpsychotic mental disorders	8.3% (1)	11.1% (1)	0% (0)
F5: Behavioural syndromes associated with physiological disturbances and physical factors	0% (0)	0% (0)	0% (0)
F6: Disorders of adult personality and behavior	75.0% (9)	22.2% (2)	66.7% (2)
F7: Intellectual disabilities	16.7% (2)	22.2% (2)	0% (0)
F8: Pervasive and specific developmental disorders	16.7% (2)	11.1% (1)	0% (0)
F91: Conduct disorders	8.3% (1)	11.1% (1)	0% (0)

Percentage of cases with(out) adult ADHD refers to the cases which (had not) received the diagnosis of ADHD at least once during the observation period. Within those cases where an ADHD diagnosis had been given during adulthood, the occurrence of diagnoses refers to co-morbid diagnoses, i.e. they were only counted if they were present at the same time as the ADHD diagnosis = during at least at one assessment point in adulthood.

The most common comorbid diagnosis overall were personality disorders present in three out of four individuals, followed by substance use disorders, which were diagnosed in two thirds of the cases. For substance use disorders, it should be noted that a diagnosis can be present even after an individual has abstained from substance use over prolonged periods of time (e.g. F1x.21 currently abstinent, but in a protected environment). Indeed, active substance abuse was not involved in all cases. Similarly, not every clinician was consistent in noting an F1 diagnosis when the individual was not actively using substances. Other disorders were less common and no F2, F3, and F5 diagnoses were observed.

Interestingly, **Table 2** highlights a considerably lower incidence of personality disorders as a comorbid disorder alongside ADHD, in contrast to their general incidence within the sample. This is notable given that in three out of four instances where an ADHD diagnosis was retracted, a subsequent diagnosis of personality disorder was established. Conversely, a transition from a personality disorder diagnosis to adult ADHD was observed in only one case. This patient’s personality disorder diagnosis was rescinded following the diagnosis of adult ADHD, a transition not evident in the remaining two cases. In the fourth case, an ADHD diagnosis was exclusively determined during an ADHD assessment, with no discussion of alternative diagnoses.

## 4 Discussion

The aim of our study was to map trajectories of childhood ADHD diagnoses in criminal offenders who received mandated treatment in a forensic outpatient clinic. In our study, 6.6% (*N* = 12) of all cases had an established diagnosis of ADHD during childhood/adolescence. Whilst this falls within the 2-7% range of prevalence rates for ADHD amongst children and adolescents typically observed (35), it is less than what we would expect in a forensic sample; meta-analytic findings suggest a prevalence estimate of up to 41% for retrospective assessments of ADHD in childhood in offenders (10). The low prevalence rate of childhood ADHD in our sample could be the result of undiagnosed childhood ADHD, or ADHD being diagnosed but not reported by the patient or not (sufficiently) documented in the files available to the expert witness. Indeed, a study which assessed forensic outpatients who were suspected of having ADHD in a standardized way found that the diagnosis had previously been missed in life in over half of the sample (25). Similar results with even larger proportions of missed diagnoses were also reported for incarcerated individuals (26, 27). Equally, forensic samples often contain an overrepresentation of individuals from socioeconomically disadvantaged backgrounds, where the potential lower availability of psychiatric or psychological care may also have contributed to the lower prevalence rates of ADHD observed in these groups (36, 37).

Of those cases where childhood ADHD was ascertained, 75% had a diagnosis of ADHD during adulthood at least one point of observation. This corresponds to roughly 5% of the overall sample, which is higher than the prevalence rate of 2% recently observed in a Swiss prison (24), albeit across a smaller sample size in our study. Likewise, it should be noted that the specific rate will depend on the time of observation, with higher rates of adult ADHD being more likely at an earlier age with an observation period during early adulthood, as opposed to a later point in time. That said, overall, our results are consistent with previous research showing how ADHD is underdiagnosed in adult offenders (24).

Within our data, the individual trajectories revealed four distinct patterns, namely: *maintained* (1 case)*, initially maintained* (4 cases)*, intermittently discontinued* (4 cases), and *discontinued* (3 cases). In one case (8%), the diagnosis was maintained throughout all assessment points during the observation period. Given the higher prevalence rates observed in adult offender populations, the proportion of persistent ADHD seems comparatively low. In a total of five cases (42%), the diagnosis was maintained until at least the age of 23. If we include those cases where the diagnosis was present at some points but not at others, the rates increase to 75% (i.e., 9 out of 12 cases), which aligns more with current estimates about the persistence of ADHD into adulthood (5–7).

Given these findings, an important question remains as to why the diagnosis is not maintained in a consistent way in two thirds of the cases (i.e., those trajectories *initially maintained* and those *intermittently discontinued*). For *initially maintained diagnoses*, the diagnosis was made at the first assessment point (an expert witness assessment) but subsequently discontinued. Conceivably, it is possible that these cases experienced symptom remission to an extent where the individuals no longer met diagnostic criteria for ADHD. Interestingly, however, the reverse pattern was apparent too. For the instances of *intermittently discontinued diagnoses*, the diagnosis was not continued during the first (and in most cases, later) points of assessment but assigned during a future stage. According to other research, ‘fluctuating’ periods of remission and recurrence over time may actually, depending on the study, be an occasional to common occurrence in individuals with ADHD (6, 38, 39); however, these studies did all focus on transition periods rather than adulthood trajectories. Despite this, preliminary evidence shows so-called ‘unstable’ pathways in adults, with a study in a non-offending population suggesting around 26% of adults with ADHD may exhibit this phenomenon (38, 40).

Amongst other factors, the idea that socioenvironmental determinants can result in periods during which the individual can cope (i.e., low symptomatology) vs. is overwhelmed (i.e., high symptomatology) seems plausible for all age groups and may be particularly relevant for offender groups [see also (41)]. Specifically, highly structured environments, such as prisons, may inadvertently contribute to a perceived reduction in ADHD symptoms amongst offenders with a previous ADHD diagnosis (42). Indeed, for some patients in our analysis, diagnoses of ADHD tended to be omitted during periods of incarceration, only to be re-evaluated upon transition to less structured probationary settings. This aligns with anecdotal evidence in the literature, wherein individuals with ADHD may exhibit “model behavior” in prison due to the benefit derived from a structured routine (42). Encompassing rigid schedules and regulations, prisons may provide the external organization needed by individuals with ADHD to function more effectively, possibly masking typical symptoms. In this regard, studies highlight a significant challenge in the recurrence of ADHD in patients transitioning from prison life after their release, highlighting an inability to self-impose the structured routines they experienced whilst incarcerated (43, 44). In our findings, this might have led to the demasking of ADHD symptoms, prompting a re-evaluation of the disorder. Nevertheless, it is important to acknowledge that the notion of the “model prison inmate”, particularly in the context of individuals with ADHD, is not without controversy. There is evidence to suggest that, despite the external constraints of the prison environment, affected individuals still exhibit significant symptomatology (45).

Another point warranting discussion is the potential differences in the diagnostic approaches both between and among professions (e.g. expert witnesses, therapists). As part of psychometric testing for ADHD assessments analyzed in the study at hand, the diagnostic tool HASE (32, 33) was used on a regular basis. Expert witnesses or therapists initiated these assessments. However, some expert witnesses and also therapists tended to base their decisions more on clinical impressions and the presence or absence of ICD criteria, although treatment reports in particular often lacked an in-depth discussion of how a verdict regarding a psychiatric diagnosis had been reached. This is notable as international guidelines for the diagnosis of ADHD highlight the importance of a comprehensive assessment process, encompassing the evaluation of biographical information and psychometric testing, alongside clinical symptomatology [e.g (46)]. According to a scoping review by Byrne and Guenter (21), prior literature indicates that diagnostic variability can impede the reliable identification and successful treatment of ADHD amongst offender samples. Given the established links between ADHD, functional deficits, and re-offending (13, 15, 16), this could conceivably have wider societal consequences in terms of recidivism and may entail detrimental outcomes for affected individuals. In sum, this underlines the need for a more holistic and standardized diagnostic approach to the assessment of ADHD in forensic settings, which ideally is adopted by the different professionals involved, as has been recommended elsewhere (23).

Other reasons for false negative ADHD diagnoses in adulthood have been outlined by Sibley (47). Notably, in adults with chronic ADHD that started in childhood, symptom under-reporting seems to be particularly common, which can impede the accurate identification of the disorder in later life (48). In the authors’ opinion, this phenomenon could be pertinent in forensic expert witness assessments where the practitioner and the patient interact only over the course of a few sessions; this is distinct from other settings where a patient is typically seen repeatedly over a prolonged period of time. Additionally, the context of forensic assessments may encourage dissimulation, as individuals might seek to avoid the stigma associated with being labelled as mentally ill offenders, further complicating the accurate diagnosis and understanding of their disorder (49). Moreover, the high comorbidities of ADHD [e.g (50–52)] may also complicate the diagnosis, with the symptoms adults experience due to their ADHD often being mistaken for those of their comorbid conditions (22). Research suggests that comorbidity rates of ADHD can be particularly high within intramural settings. A study by Einarsson et al. noted that around 96% of those with symptomatic ADHD also had at least one other comorbid psychiatric disorder (53). In our sample, in seven out of the eight cases for which this question could be analyzed, at least one comorbid psychiatric disorder was present. Equally, co-morbidity rates are higher for incarcerated people with ADHD than for incarcerated people without ADHD (11), a pattern which again was also present in our data with forensic outpatients. In sum, both these factors could contribute to ADHD being obscured by the presence of other disorders, providing possible reasons for the fluctuations we observed in the patient trajectories (54).

However, an alternative explanation for the fluctuations in adult ADHD diagnoses needs to be discussed. A striking finding in our results was that in half of the cases, in which an ADHD diagnosis was initially maintained or intermittently discontinued, a personality disorder was diagnosed once an ADHD diagnosis was ceased (3 cases), and vice versa (1 case). This could imply that similar symptoms may have been interpreted differently, depending on the specific stakeholder’s perspective. In fact, in one case, an expert witness noted that the remaining ADHD symptoms were now incorporated into the diagnosis of an emotionally instable personality disorder with accentuated dissocial traits. Similarly, another expert argued that a severe dissocial personality disorder developed on the basis of ADHD. In another case, the treatment report stated, “the newly made diagnosis for adults [personality disorder with dissocial and emotionally instable (impulsive) elements] corresponds in its presentation largely to the previous diagnosis [hyperkinetic conduct disorder], which is used for children and adolescents”.

Adult ADHD and borderline personality disorder (BPD) share some core clinical features, including impulsivity, emotional dysregulation, and cognitive impairment [see (55)]. Additionally, adult ADHD often co-occurs with BPD [e.g (56, 57)]. Storebø and Simonsen further found that children with ADHD are at an increased risk of developing personality disorders, in particular ASPD (56). Certain studies suggest that impulsivity in ADHD or callous-unemotional traits may be predictive of later ASPD [see (58)] and others highlighted high comorbidity rates between ADHD and ASPD [e.g (13, 50)]. Interestingly, in our sub-sample of those with a current ADHD diagnosis, we observed a substantially lower comorbidity rate with personality disorder than in the main sample.

This raises the question as to whether some of the expert witnesses and perhaps also some of the therapists are reluctant to award dual diagnoses. This warrants further investigation since dual diagnoses are outlined explicitly in the ICD, which is the classification manual typically used in the German-speaking part of Switzerland. It is possible that different perceptions in the professionals assessing the patients may have contributed to this fluctuation in the diagnosis. Another potential reason for the limited recognition of ADHD in this context might stem from prevailing biases within the forensic community, possibly rooted in the educational framework and content provided by Swiss forensic psychiatry and psychology training courses. Notably, the emphasis in these programs appears to be on “forensically relevant psychiatric disorders,” with a predominant focus on personality disorders and paraphilias [e.g (59–61)]. The pattern was also evident when examining the predecessors of these schemes (known as *Schwerpunkttitelkurse*), where a similar focus could be discerned. This tendency towards personality disorders at the expense of covering neurodevelopmental disorders may offer a conceivable explanation for their underrepresentation in forensic assessments. In the authors’ opinion, these limitations in the educational framework in Switzerland illustrate a need for a more inclusive curriculum that encompasses a broader spectrum of psychiatric disorders, including ADHD, to ensure comprehensive assessments in forensic psychiatry.

Should an individual not receive an adult ADHD diagnosis because of a professional’s inclination to diagnose personality disorders, despite the fact that they might meet the criteria for adult ADHD, this would of course be highly problematic. Whilst specific ADHD symptoms respond well to, amongst others, psychopharmacological treatment, no such treatment is available for personality disorders (62). Certain research findings have shown that medical treatment for ADHD in offenders can be associated with a lower risk of recidivism (18, 22). By failing to detect and adequately treat ADHD, patients may also struggle in following rehabilitative protocols and controlling impulsive behaviors, thereby impinging upon their successful reintegration. Yet, the recognition that ADHD persists throughout the lifespan and has seen a shift to a longitudinal approach in its management, as accentuated by the ADHD Life Transition Model (63), does not seem to have received extensive attention in forensic psychiatry; at the time of writing, the scarcity of publications on this in forensic-psychiatric journals suggests a gap in awareness and applications of this lifespan perspective in forensic settings, where understanding the continuity and evolution of ADHD could significantly influence patient management and outcomes. In sum, ensuring proper diagnosis and treatment of ADHD could help mitigate a number of issues, facilitate rehabilitation and contribute towards reducing reconviction rates.

In the context of this study, the presence of ADHD was not re-assessed by the research team. We cannot ascertain, therefore, whether the omission of a diagnosis of ADHD during adulthood was justified per diagnostic criteria. Separating instances of potentially fluctuating ADHD from those where the full symptoms of ADHD are no longer present or where a diagnosis was ceased incorrectly (i.e. lost) is not possible. Whether the diagnoses are lost because professionals arrive at different conclusions or whether it is related to the possible fluctuating nature of ADHD requires detailed investigation. Nonetheless, it is important to note that there are inherent limitations in the interrater reliability of diagnostic criteria, including those set forth in the ICD (64). Achieving perfect interrater reliability is not feasible, and this reality must be factored into our considerations and analyses. However, our findings raise complex questions regarding possible personal attitudes or educational biases of key stakeholders, such as forensic expert witnesses, towards adult ADHD. This is an important consideration, since forensic expert witness assessments carry considerable weight in future treatment directions; treatment providers may erroneously assume an ADHD diagnosis is no longer relevant if it is discontinued by an expert witness. Whilst, encouragingly, we have seen instances in which the reintroduction of an ADHD diagnosis was initiated by a therapist, there may be other instances where this did not transpire.

Investigating forensic expert witnesses’ attitudes towards adult ADHD is challenging, as demand characteristics and socially desirable responding are likely to distort responses. One possibility might be to analyze forensic expert witness reports of individuals with childhood ADHD more closely regarding the points of discussion raised and justifications presented if the diagnosis is discontinued. This could provide indirect insight into experts’ perceptions of adult ADHD. In short, although general awareness about ADHD is increasing, there remains a lack of education and therapeutic implementation for adults with this condition in general (65) and, specifically, also in for forensic populations. Finally, another issue that should be considered in future research is residual symptomatology. In more than one instance, we observed that residual symptoms were discussed, which were deemed insufficient to warrant a diagnosis. Still, in some cases, psychopharmacological treatment was put or kept in place. More studies on the potential use (vs. the potential costs) of psychopharmacological treatment in cases with strong residual symptoms that fail to reach the criteria for full diagnoses are therefore needed.

## 5 Study strengths and limitations

We assessed the full sample of concluded cases at one of the largest former Swiss forensic outpatient clinics (BFOC) and mapped the trajectory of adulthood ADHD in 12 offenders per our inclusion criteria. Whilst this limited sample size was not surprising given the known underdiagnosis of ADHD in offender groups, it restricted potential data analyses. Nevertheless, the findings provide important new hypotheses and future research directions regarding possible trajectories in adult ADHD amongst forensic samples; specifically, this includes where and why problems might occur that prevent patients with ADHD from receiving the proper diagnosis and treatment.

In forensic contexts, retrospective file analysis has been employed elsewhere [e.g (66, 67)] and in our study, this approach provided promising avenues to explore adult trajectories in offender samples. This is particularly pertinent since Swiss forensic expert witness assessments are well known for the wealth of information and their comprehensiveness with regards to the examinee. Usually, forensic expert witnesses have access to detailed files and medical records of their patients, which enabled us to analyze a large volume of cases and filter those that required closer examination. Although there is a degree of uncertainty regarding the completeness of the available information, we were able to gather a comprehensive picture of individual cases. That said, there will likely be missing assessment points for some of the patients. For instance, not all treatment reports from other institutions were available and, thus, we could not code these perspectives about patient diagnoses. Additionally, this study used a keyword search to identify relevant ADHD diagnoses in lieu of manually scanning each document. Accordingly, despite the fact that comprehensive keywords were adopted, we cannot rule out that passages may have been missed during this process. Likewise, the conversion of scans into readable PDF files may have resulted in poor resolution or included annotations to the original documents in certain passages, possibly affecting the search. Yet, this approach enabled us to search a very large volume of documents, which would not have been feasible through other means.

The inclusion criterion was restrictive in the sense that it only included fully established ADHD diagnoses, which was designed to ensure the validity of the data. However, in some cases, persisting symptoms of ADHD were discussed, e.g. in an expert witness assessment or treatment report, but as the symptoms were considered to be below the threshold of a diagnosis, no adult ADHD diagnosis was made. Interestingly, we noticed that in some of these cases, patients received medical treatment for their ADHD symptoms, despite not having a validated ADHD diagnoses. Moreover, we were only interested in the trajectories of those with an established diagnosis of ADHD in childhood or adolescence. This approach circumvented the problems associated with missing the diagnosis during childhood and the complications that may arise when trying to retrospectively assess childhood ADHD as an entry criterion for adult ADHD. In that sense, we created an optimal starting position for the providers involved in assessing these patients as adults. This means, however, that we did not include any cases where an individual received the diagnosis of ADHD for the first-time during adulthood. Thus, the prevalence rate of adult ADHD is conservative at best and could provide an interesting topic for future investigations.

Despite these limitations, our results make a valuable contribution to the wider topic of ADHD in offender populations, especially within Switzerland’s criminal justice system. Equally, we believe that our study provides a basis for more detailed longitudinal investigations in this area and raises important issues related to the underdiagnosis and lack of attention towards ADHD in offender samples and beyond.

## 6 Conclusion

The results from this study suggest considerable inconsistency in how expert witnesses and therapists address adult ADHD in individuals with a diagnosis of childhood/adolescent ADHD. In existing literature, it is well-established that for a considerable proportion of individuals with childhood ADHD, symptoms persist into adulthood with related functional impairments. Yet, it is not clear whether this knowledge-base has reached forensic-psychiatric stakeholders. The call to educate and sensitize prison staff towards ADHD should be bolstered by similar initiatives tailored to forensic-psychiatric expert witnesses and therapists (23). Although, based on available data, the research team was not in a position to determine whether an adult ADHD diagnosis is still warranted in individual cases and the potential role of fluctuating ADHD needs to be considered, the large number of ultimately discontinued ADHD diagnoses prompts concerns. It is questionable whether changing an ADHD diagnosis to a personality disorder diagnosis based on a number of shared symptoms when, for the former, various empirically supported treatment options are available whereas options are limited for the latter is guided by best practice. In sum, our findings highlight a need for further research on the education and decision-making processes of forensic-psychiatric practitioners in relation to potential cases of adult ADHD. Additionally, more research on the effectiveness of treating persisting symptoms that are below the threshold of an ADHD diagnosis in a forensic-psychiatric context could inform tailored considerations for reducing the risk of recidivism and improving individual mental health.

## Data availability statement

The raw data supporting the conclusions of this article will be made available by the authors, without undue reservation.

## Ethics statement

The studies involving humans were approved by the Ethics Commission of the Canton of Bern (ref.-no. 2022-02113). The studies were conducted in accordance with the local legislation and institutional requirements. Written informed consent for participation was not required from the participants or the participants’ legal guardians/next of kin in accordance with the national legislation and institutional requirements.

## Author contributions

HW: Conceptualization, Formal analysis, Investigation, Methodology, Validation, Visualization, Writing – original draft, Writing – review & editing, Data curation, Supervision. MvW: Conceptualization, Visualization, Writing – original draft, Writing – review & editing, Data curation, Formal analysis, Methodology. AS: Conceptualization, Visualization, Writing – original draft, Writing – review & editing. WR: Writing – review & editing. ML: Conceptualization, Methodology, Project administration, Resources, Supervision, Writing – original draft, Writing – review & editing, Validation. AB: Conceptualization, Methodology, Supervision, Writing – review & editing.
