# Supplementary material for: Lost diagnoses? A multi-year trajectory of patients with childhood ADHD in the criminal justice system in Switzerland
Source: Front Psychiatry. 2024 Jun 6;15:1403618. doi: 10.3389/fpsyt.2024.1403618 (PMC11187301; doi:10.3389/fpsyt.2024.1403618)
Supplement: Supplementary file 1 [file DataSheet_1.docx]

**Supplemental Material -- Comorbidities identified throughout the patient trajectories**

A indicates an ADHD assessment, E refers to a forensic-psychiatric expert witness report, and all other assessment points are based on treatment reports. For the avoidance of doubt, all diagnoses classifications are based on ICD-10. All filled points indicate the presence of a specific diagnosis. For empty points, a specific diagnosis was not made. PX refers to the individual patient cases also referred to in the results section.

*Diagnosis Maintained*

P1


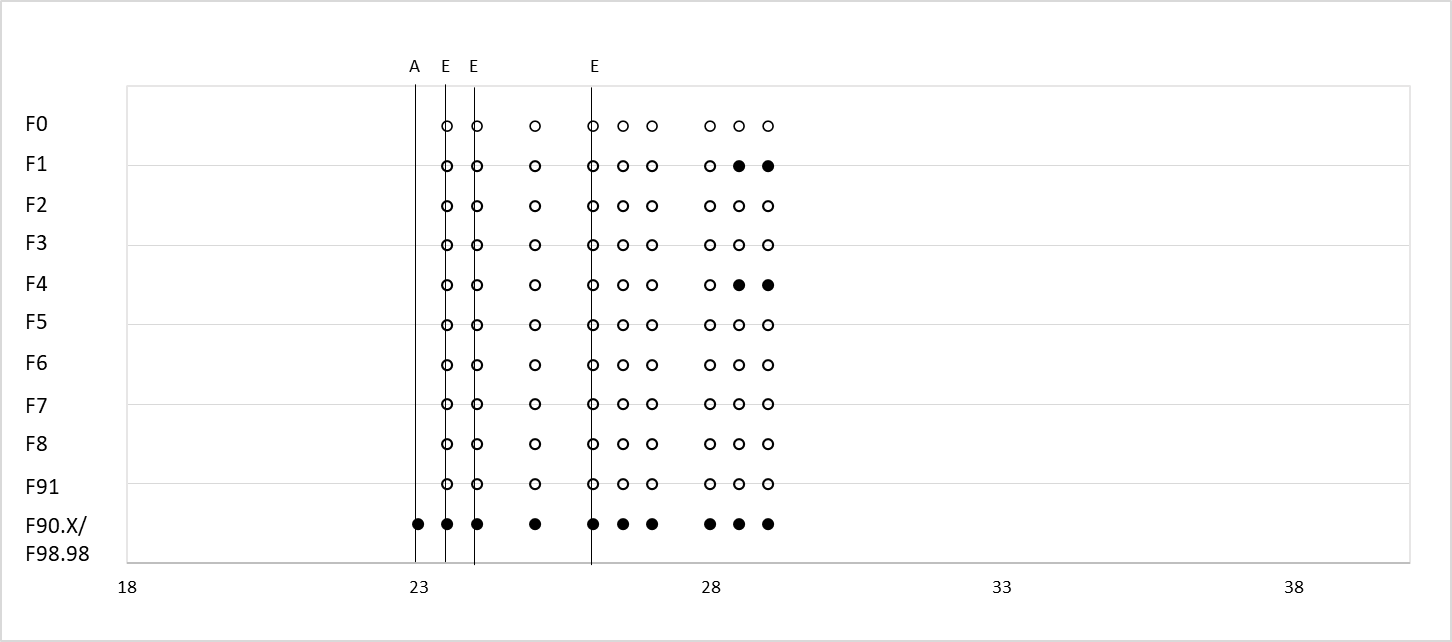


*Diagnosis Initially Maintained*

P2

*
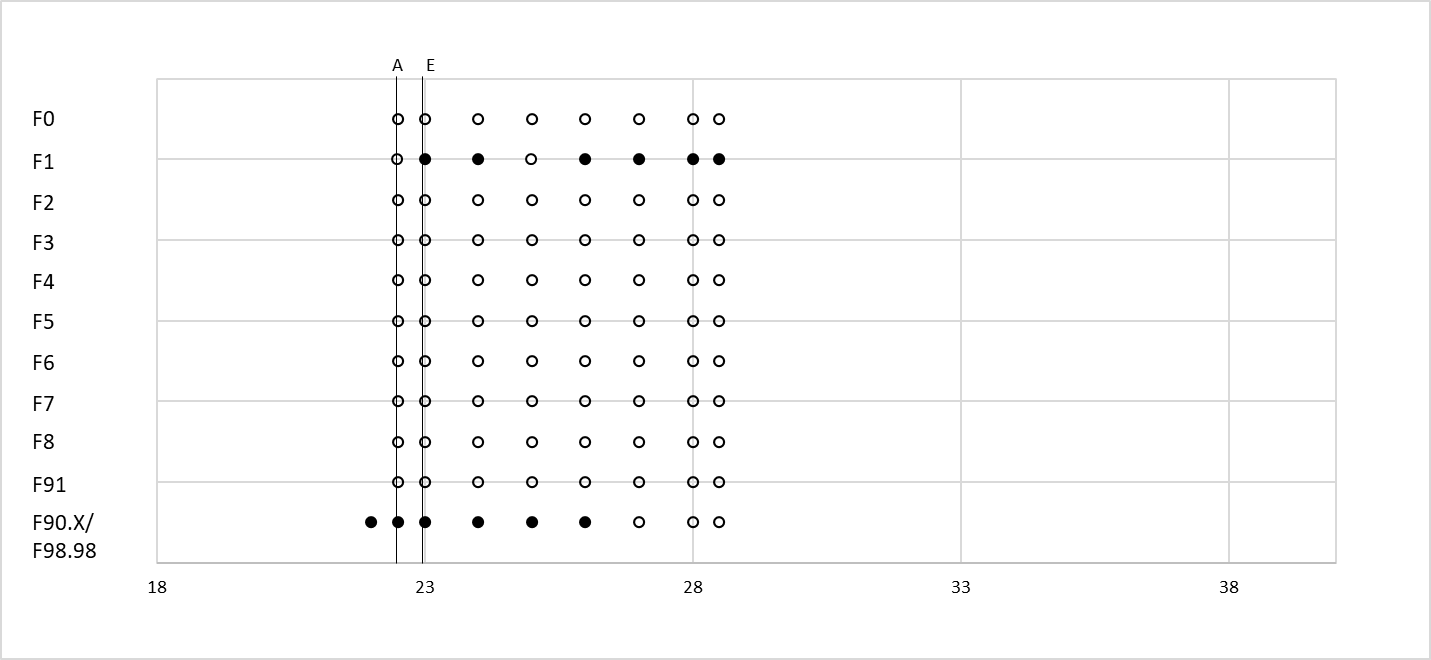
*

P3


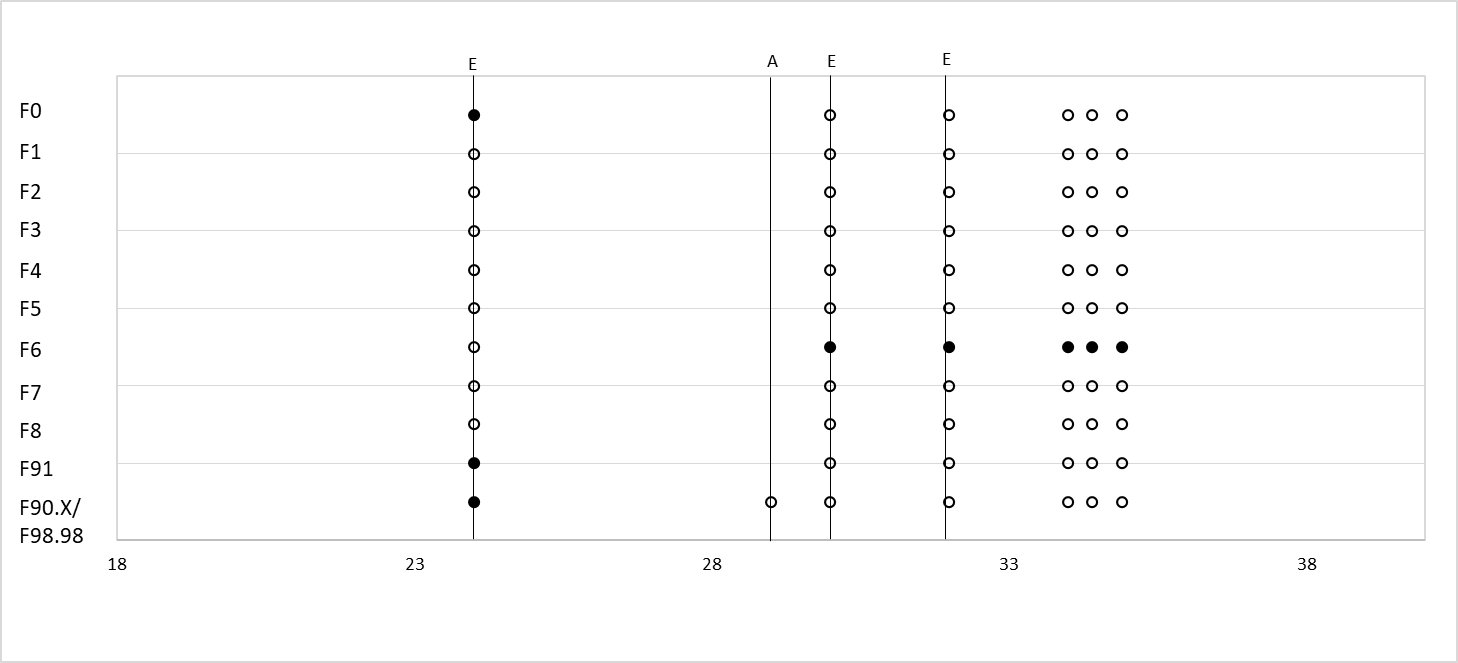


P4


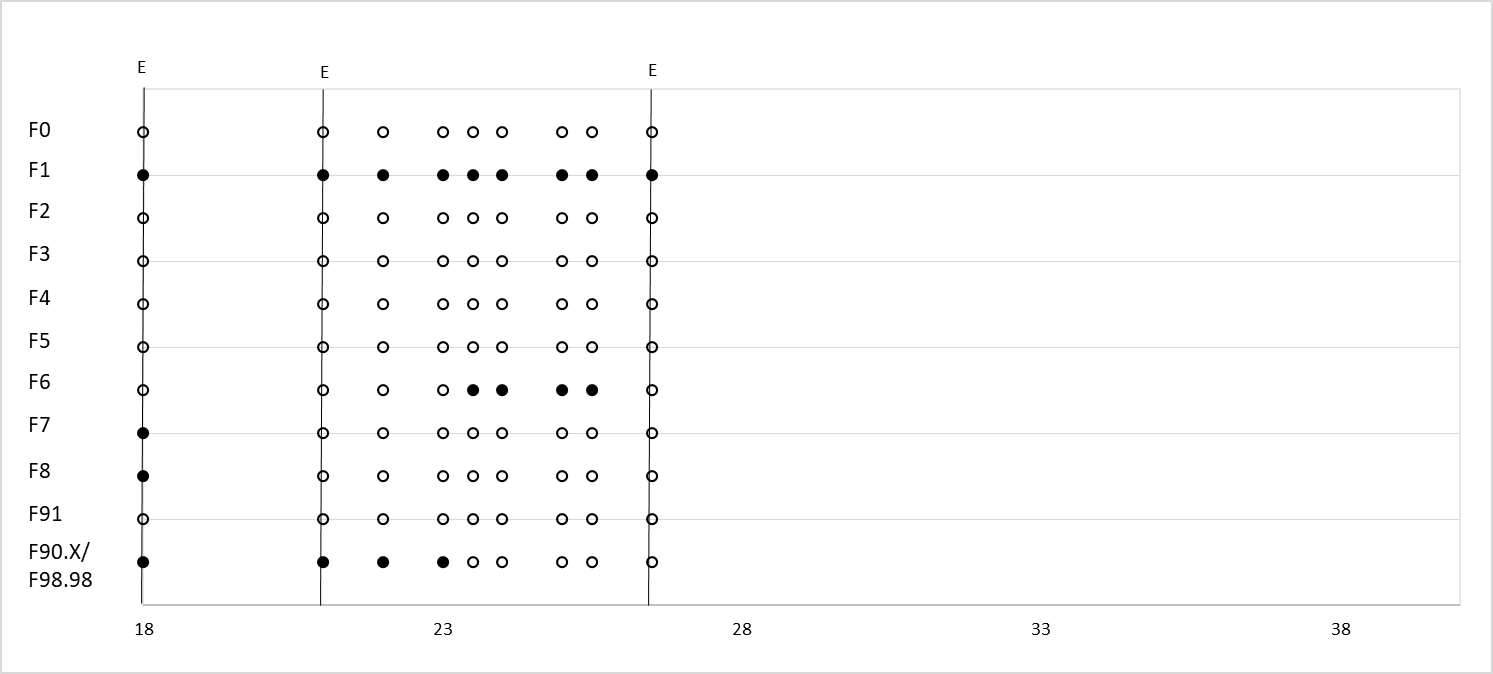


P5


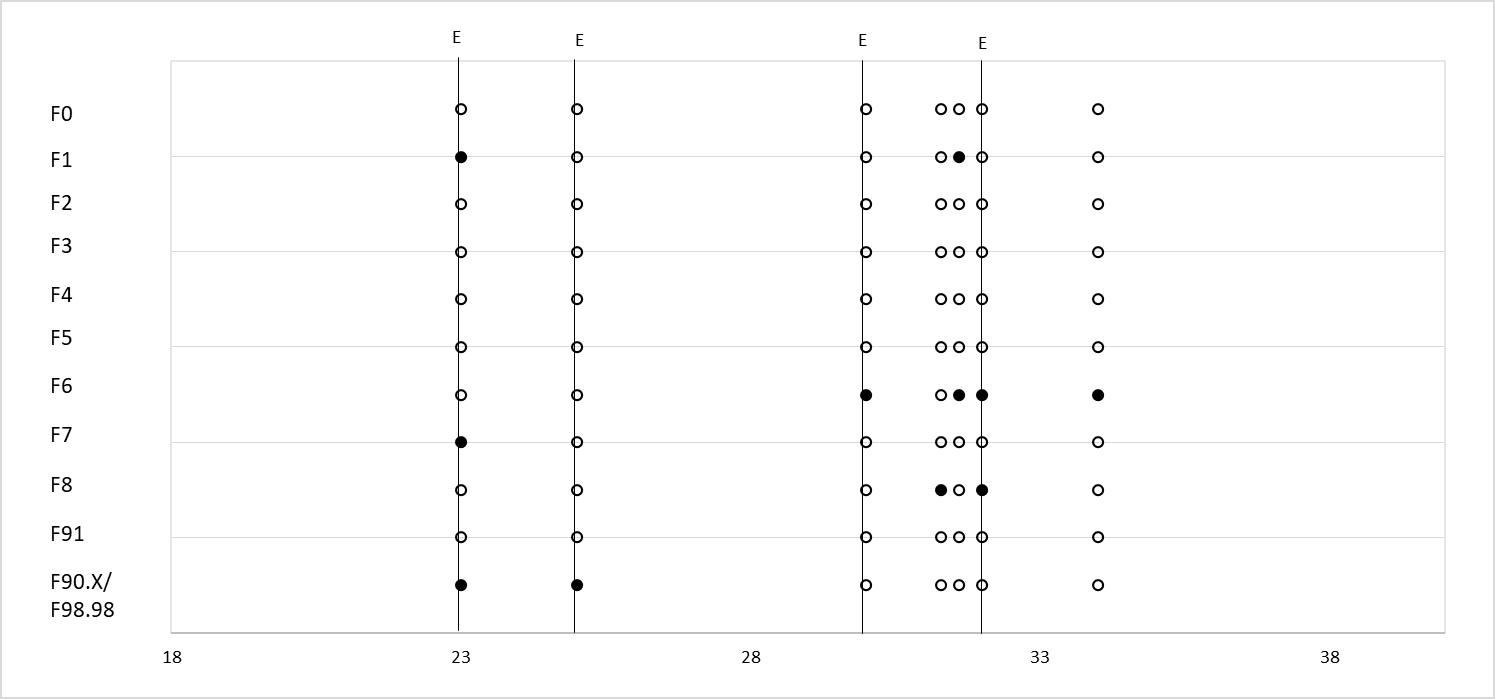


*Diagnosis Intermittently Discontinued*

P6

*
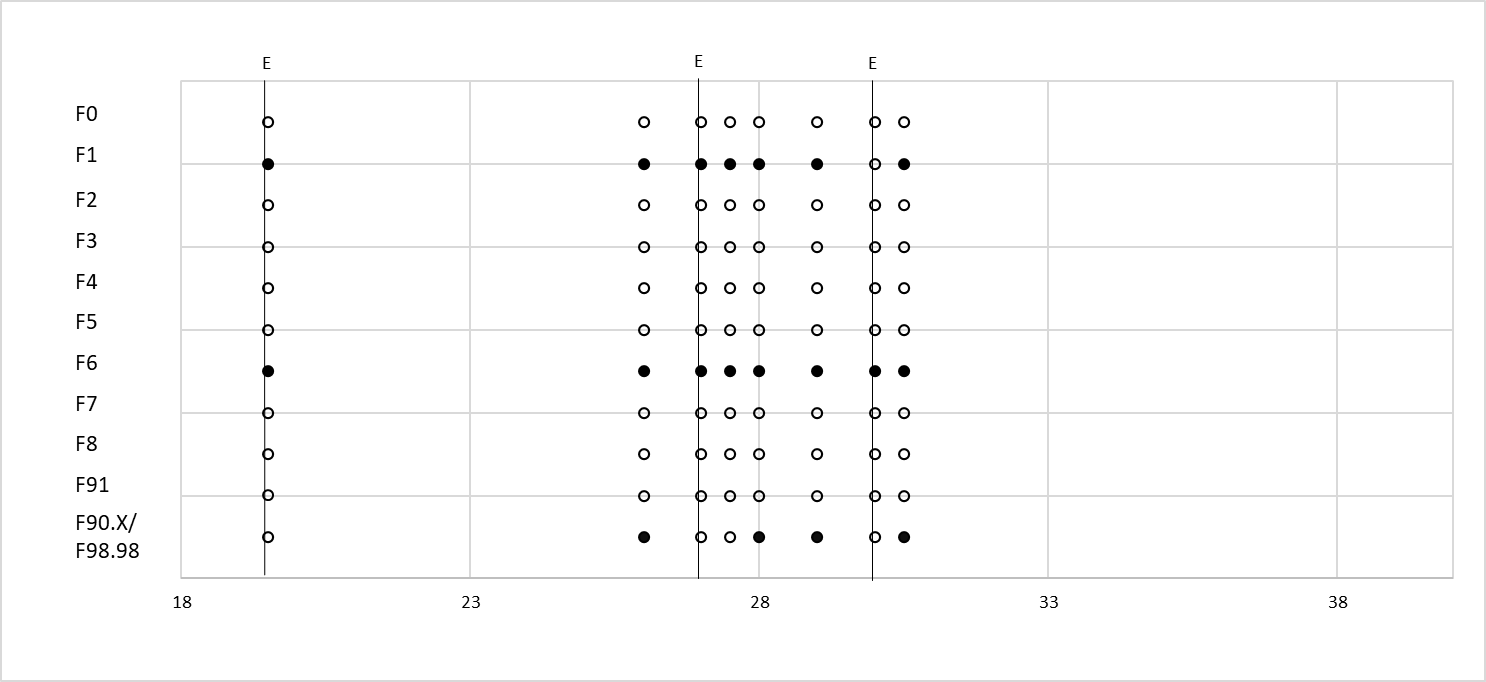
*

*
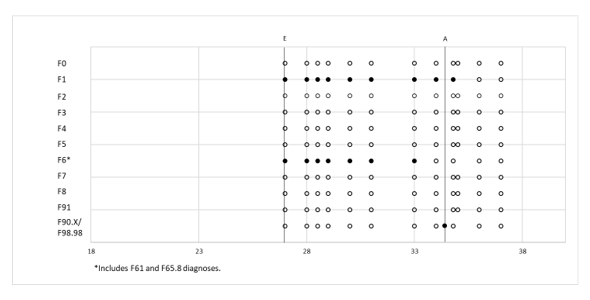
*P7

P8

*
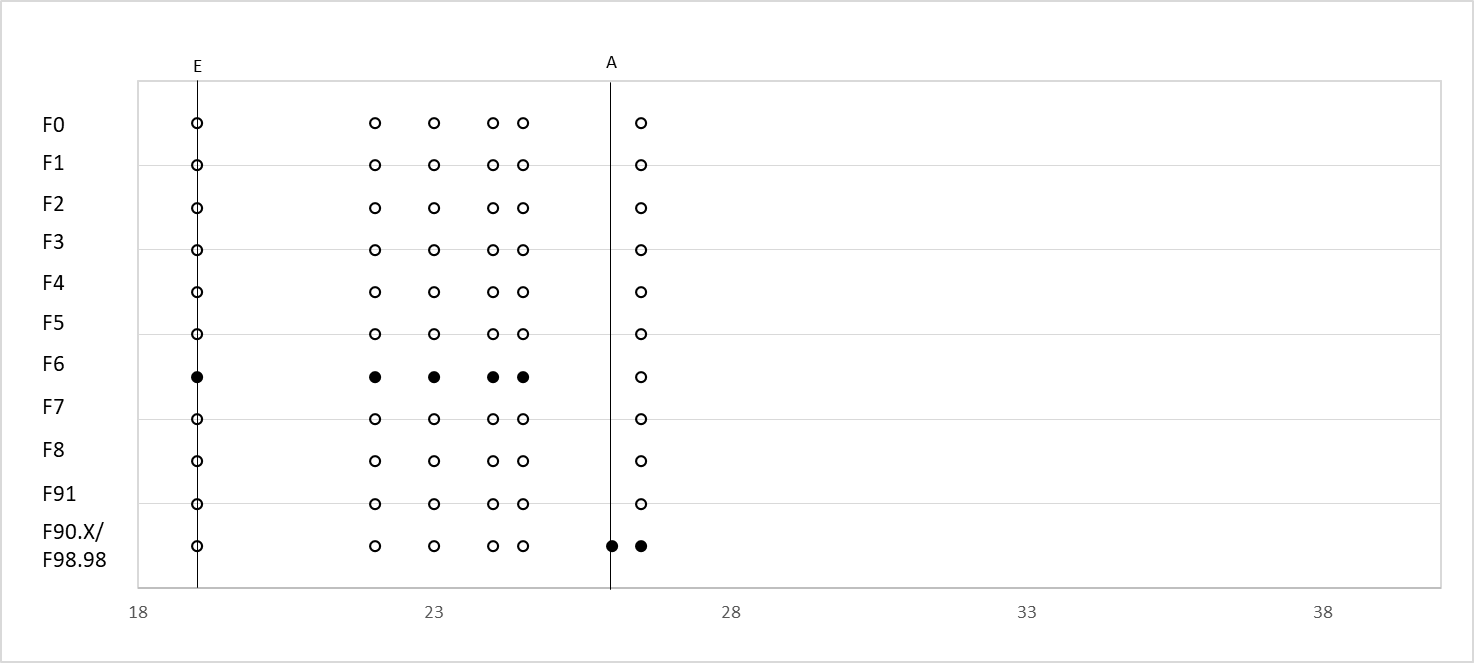
*

P9

*
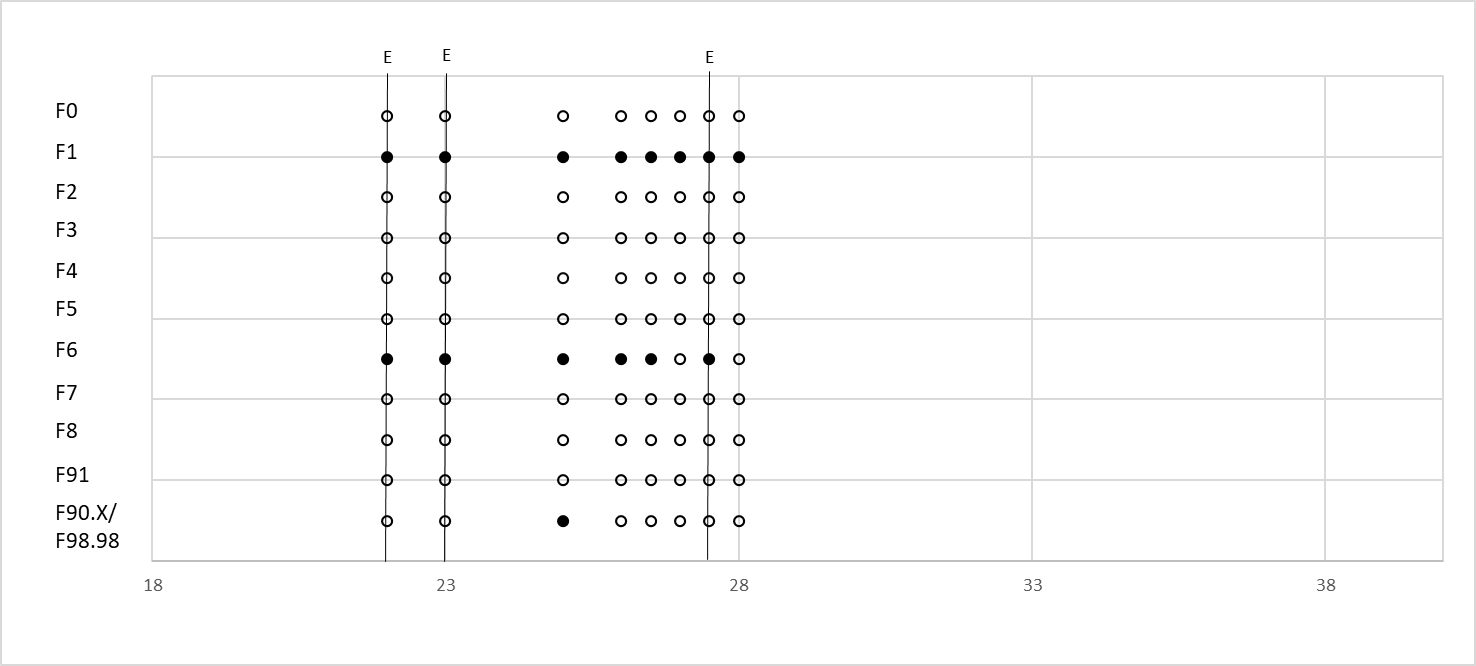
*

*Diagnosis Discontinued*

P10

*
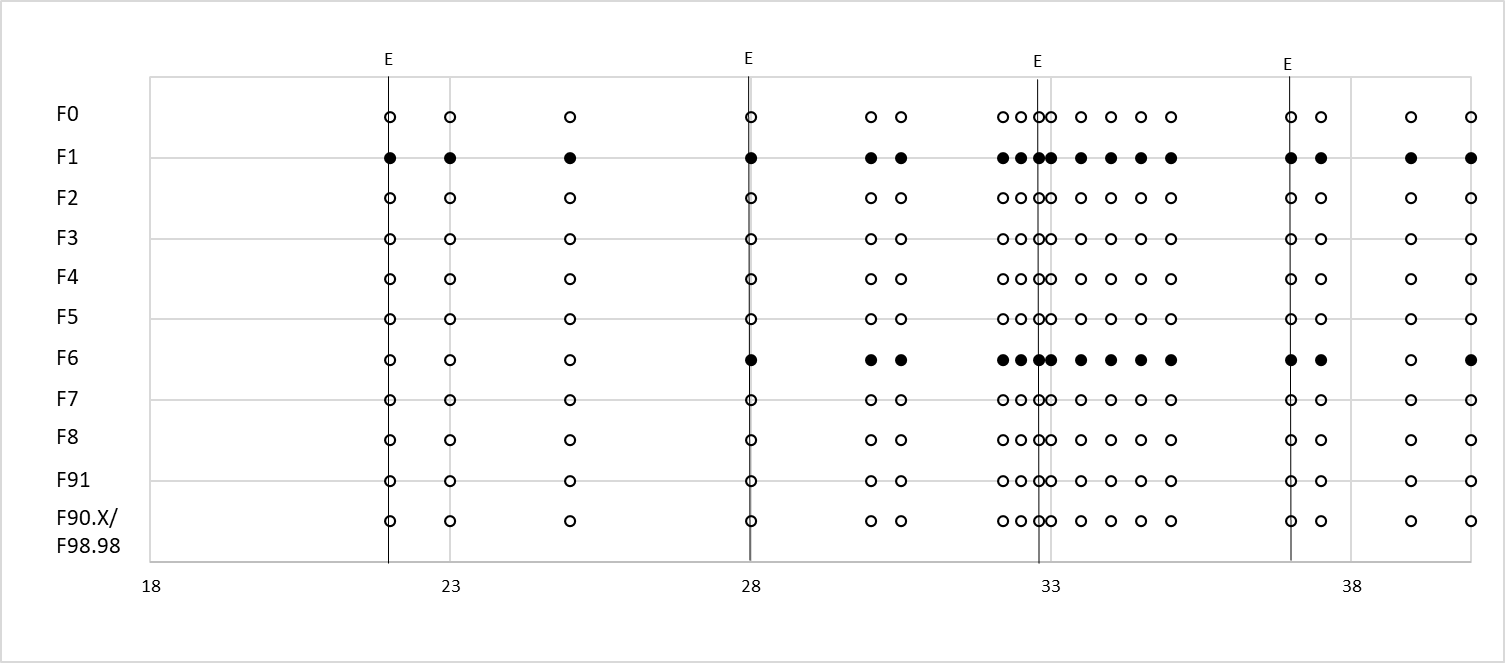
*

P11

*
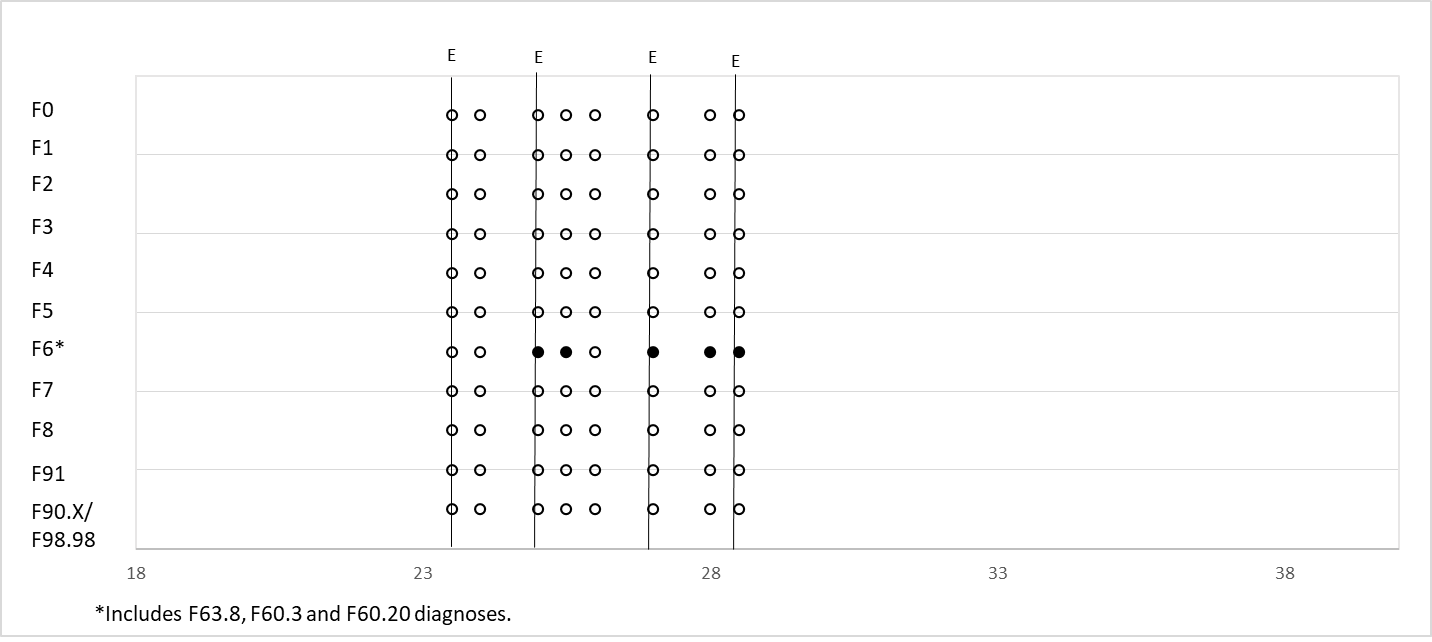
*

P12

*
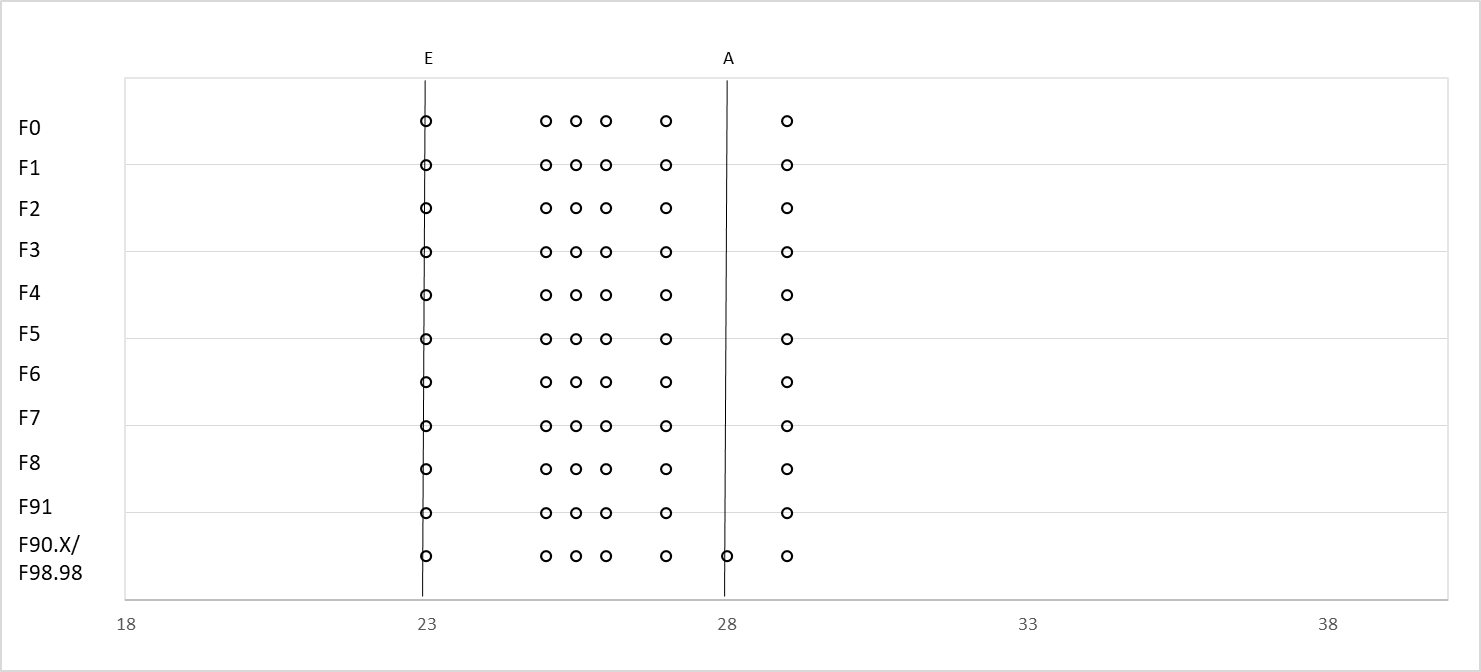
*
